# KCNMB2-AS1 Promotes Bladder Cancer Progression Through Sponging miR-374a-3p to Upregulate S100A10

**DOI:** 10.3389/fgene.2021.655569

**Published:** 2021-07-22

**Authors:** Jianhua Zhu, Yan Huang, Yong Zhang, Rongfu Huang, Chunmei Huang

**Affiliations:** ^1^Laboratory of Clinical Immunology, Wuhan No.1 Hospital, Tongji Medical College, Huazhong University of Science and Technology, Wuhan, China; ^2^Department of Orthopedics, Tongji Hospital, Tongji Medical College, Huazhong University of Science and Technology, Wuhan, China; ^3^Department of Clinical Laboratory, The Second Affiliated Hospital, Fujian Medical University, Quanzhou, China; ^4^Department of Pathology, The Central Hospital of Wuhan, Tongji Medical College, Huazhong University of Science and Technology, Wuhan, China

**Keywords:** bladder cancer, lncRNA KCNMB2-AS1, S100A10, proliferation, migration, invasion, miR-374a-3p

## Abstract

Long non-coding RNAs (lncRNAs) have been reported to play a crucial role in the pathogenesis of numerous cancers. However, the function of lncRNA KCNMB2-AS1 in bladder cancer (BC) remains unclear. In the present study, we aimed to explore the role and underlying mechanisms of KCNMB2-AS1 in bladder cancer progression. We found that lncRNA KCNMB2-AS1 was significantly upregulated both in BC tissues and cell lines, the expression level was highly correlated with pathological TNM stage. Functionally, knockdown of lncRNA KCNMB2-AS1 dramatically inhibited the proliferation, migration, and invasion and of BC cells *in vitro*, and suppressed tumor growth *in vivo*. Mechanistically, lncRNA KCNMB2-AS1 could function as a competitive endogenous RNA (ceRNA) through direct sponging miR-374a-3p, which regulated the expression of S100A10. In conclusion, our results demonstrated that lncRNA KCNMB2-AS1 can promote the progression of bladder cancer through regulation of miR-374a-3p/S100A10.

## Introduction

Bladder cancer (BC) is one of the most common malignant tumors of the urinary system (Li W. et al., 2020). The incidence and mortality of BC is increasing steadily (Chen et al., 2016). Although surgery and chemotherapy methods have improved, the 5-year survival rate remains unsatisfactory. Therefore, much effort should be made to explore new diagnostic markers and therapeutic targets to improve the diagnosis and treatment of BC patients.

Long non-coding RNAs (LncRNAs) are a type of non-coding RNAs, which are more than 200 bp in length. Recently, cumulative evidence has revealed that lncRNAs play important roles in various cancers (Lian et al., 2019; Chen et al., 2020; Xie et al., 2020). Many lncRNAs have been identified and wield significant influence in the progression of bladder cancer. For instance, KCNQ1OT1 promotes the progression of bladder cancer by regulating miR-218-5p/HS3ST3B1 axis (Li Y. et al., 2020). LncRNA CASC11 has been reported to promote bladder cancer cell proliferation (Luo et al., 2019). GAS6-AS2 acts as a ceRNA to promote the proliferation and metastasis of bladder cancer cells (Rui et al., 2019). KCNMB2-AS1 has been revealed to promote cervical cancer tumorigenesis through regulating miR-130b-5p/miR-4294/IGF2BP3 (Zhang Y. et al., 2020). However, the influence of KCNMB2-AS1 on bladder cancer remains unknown.

In the present study, we demonstrated that KCNMB2-AS1 played a vital role in the progression of BC *via* miR-374a-3p/S100A10 axis. Our research broadens the insights into the underlying mechanisms in BC cell progression and provides a novel therapeutic target for BC treatment.

## Materials and Methods

### Clinical Tissue Sample

A total of 42 bladder cancer patients tumor tissues and paired adjacent non-cancer tissues were collected from Wuhan No.1 Hospital, Affiliated to Huazhong University of Science and Technology. All patients signed informed consent, this study was approved by the Ethics Committee of Wuhan No.1 Hospital.

### Cell Culture and Transfection

Bladder cancer cell lines (T24, J82, 5,637, and HT-1376) and normal urothelial cell line SV-HUC-1 were purchased from the Shanghai Institute of Cell Biology at the Chinese Academy of Sciences (Shanghai, China). Cells were cultured in RPMI 1640 medium (Invitrogen, Carlsbad, CA, United States) supplemented with 10% fetal bovine serum (FBS, Invitrogen) in a humidified atmosphere containing 5% CO_2_ at 37°C.

### shRNA Interference and Overexpression Plasmids Construction

The shRNAs sequence with specifically interfering effect (sh-KCNMB2-AS1) and negative control shRNA (sh-NC) were synthesized by GenePharma (Shanghai, China). Overexpression of S100A10 (OE-S100A10) and the empty vector (Vector), miR-374a-3p mimics, negative control (NC) mimics, miR-374a-3p inhibitor and NC inhibitor were obtained from GenePharma. Cell transfections were conducted by using Lipofectamine 2000 Reagent (Life Technologies Corporation, Carlsbad, CA, United States) according to the manufacturer’s instructions.

### Quantitative Real-Time PCR

Total RNA was extracted from tissues or cells using TRIzol Reagent (Invitrogen, Thermo Fisher Scientific, Inc., Waltham, MA, United States). The Revert Aid First Strand cDNA Synthesis Kit (Thermo Fisher Scientific, Waltham, MA, United States) was used to synthesize Complementary DNAs from total RNA. qRT-PCR was analyzed using the SYBR Green PCR Master Mix Kit (Takara Biotechnology Co., Ltd., Dalian, China) in Biosystems 7500 Sequence Detection System (Applied Biosystems). The relative expression was normalized to glyceraldehyde 3-phosphate dehydrogenase (GAPDH) or U6 and calculated according to the 2^–Δ^ ^Δ^ Ct method. The primer sequences were listed as follows: KCNMB2-AS1 (forward: 5′-TCCAACACTCCAGTGGCATC-3′ and reverse: 5′-AAGGCTGACCCACACTGTTT-3′). miR-374a-3p (forward: 5′-CUUAUCAGAUUGUAUUGUAAUU-3′ and reverse: 5′-AAUUACAAUACAAUCUGAUAAG-3′); S100A10 (forward: 5′-AAAGACCCTCTGGCTGTGG-3′ and reverse: 5′-AATCCTTCTATGGGGGAAGC-3′); GAPDH (forward: 5′ -GGATTTGGTCGTATTGGGCG-3′ and reverse: 5′-CGGTGCC ATGGAATTTGCC-3′); U6 forward: 5′-CTCGCTTCGGCA GCACA-3′ and reverse: 5′-AACGCTTCACGAATTTGCGT-3′).

### Subcellular Fractionation Assay

The cytoplasmic and nuclear extracts were extracted from bladder cancer cells with NE-PER Nuclear and Cytoplasmic Extraction Reagents (Thermo Fisher Scientific, Waltham, MA, United States). Then the relative expression of lncKCNMB2-AS1 was determined by qRT-PCR. The expression of U6 in the nucleus, and GAPDH in the cytoplasm was used as a control.

### RNA Fluorescence *in situ* Hybridization

Specific fluorescence-conjugated probes were designed by Life Technologies (Shanghai). The signals of the probe were detected by FISH Kit (GenePharma, Shanghai) according to the manufacturer’s instructions. Nuclei were stained with DAPI. The images were monitored and captured by confocal microscopy (Leica Microsystems, Mannheim, Germany).

### Cell Counting Kit-8

The cell proliferation was assessed by Cell Counting Kit-8 (CCK-8) assay (DoJinDo, Shiga, Japan). The transfected cells were (5 × 10^3^ cells per well) were plated into 96 well plates. After incubation for 0, 24, 48, and 72 h, 10 μl of CCK-8 solution was added and incubated for 2 h. The absorbance was determined at a wavelength of 450 nm.

### Colony Formation Assay

Cells (1 × 10^3^ cells per well) were plated into 6 well plates. After being cultured for 14 days, cell colonies were fixed for 30 min, then stained with 0.5% crystal violet.

### Transwell Migration and Invasion Assays

For transwell assay, a transwell chamber (EMD Millipore, Billerica, MA, United States) with or without precoated matrigel (BD Science, United States) was used to assess the migration and invasion of cells. 2.5 × 10^4^ cells were seeded in a transwell chamber with 200 μL serum-free medium. 500 μL medium containing 10% FBS was added to the lower chamber. The 24-well chambers were then incubated at 37°C for 24 h, the cells on the lower surface of the membrane were fixed in 4% paraformaldehyde for 30 min and stained with 0.5% crystal violet solution. The transmembrane cells were observed and counted under a microscope and five fields were randomly selected.

### Western Blot Analysis

Total proteins were prepared with RIPA lysis buffer (Beyotime, China). Concentrations of protein were examined by BCA assay. Equal amounts of protein were separated by 10% SDS-PAGE and electrotransferred onto polyvinylidene difluoride membranes (Bio-Rad, United States). After being blocked using 5% non-fat milk for 1 h at room temperature, the primary rabbit anti-human antibodies against S100A10 (ab76472, Abcam) and GAPDH (ab9485, Abcam) were supplemented overnight at 4°C. Then the members were washed with tris-buffered saline Tween 20 for three times and probed with horse radish peroxidase-conjugated secondary antibodies (ab205718, Abcam) for 1 h at 37°C. The bands were visualized using a ChemicDocXRS system (Bio-Rad, United States).

### Luciferase Reporter Assay

For luciferase reporter assay, The wild-type and mutant binding sites of miR-374a-3p in KCNMB2-AS1 sequence or S100A10 3′UTR were sub-cloned into pmirGLO dual-luciferase vector (Promega, Madison, WI, United States). Afterward, these vectors were co-transfected with miR-374a-3p mimics or its negative control into BC cells. 48 h later, The relative luciferase activity was measured with the Dual-Luciferase Reporter Assay System (Promega, China).

### RNA Immunoprecipitation Assay

RNA Immunoprecipitation (RIP) assay was carried out for investigating the potential interaction by using the EZMagna RIP kit (Millipore, Bedford, MA, United States) according to the manufacturer’s instructions. Briefly, anti-Ago2 antibody (ab57113, Abcam) or control anti-IgG (ab131368, Abcam) were conjugated to magnetic beads and were incubated with the cell extract in RIP buffer. The relative expression of KCNMB2-AS1 and miR-374a-3p was measured by qRT-PCR.

### Xenograft Mouse Model

All experimental and animal care procedures were approved by the Animal Research Ethics Committee of Wuhan No.1 Hospital. Nude mice (female BALB/c-nu, 4 weeks old) were obtained from the National Laboratory Animal Center (Beijing, China). T24 cells (5 × 10^6^) stably transfected with sh-KCNMB2-AS1 or sh-NC were injected subcutaneously into the left flank of nude mice. The volume of the tumors was measured every week after implantation. The tumor volume was calculated by the following formula: tumor volume (mm^3^) = (length) × (width)^2^ × 0.5. The mice were sacrificed after 35 days. The tumors were excised, and the tumor weight was measured.

### Immunohistochemistry

Tissue sections were prepared and subjected to immunohistochemical analysis. Anti-human Ki67 (ab15580, Abcam), S100A10 (ab76472, Abcam) antibody was used as primary antibodies. HRP-conjugated secondary Ab was used as secondary antibody. The images were captured by Olympus-BX51 microscope (Olympus, Japan).

### Statistical Analysis

SPSS 21.0 software (SPSS, Chicago, IL, United States) was adopted for statistical analysis. Data were presented as the mean ± standard deviation of three independent experiments. The student’s *t* test was adopted for the differences between two groups. Comparisons between multiple groups were performed using one-way ANOVA. Cell viability at different time points was compared using two-way ANOVA. Gene expression correlation was conducted by Pearson’s correlation analysis. ^∗^*P* < 0.05 was considered statistically significant.

## Results

### KCNMB2-AS1 Was Upregulated in BC

**FIGURE 1:**
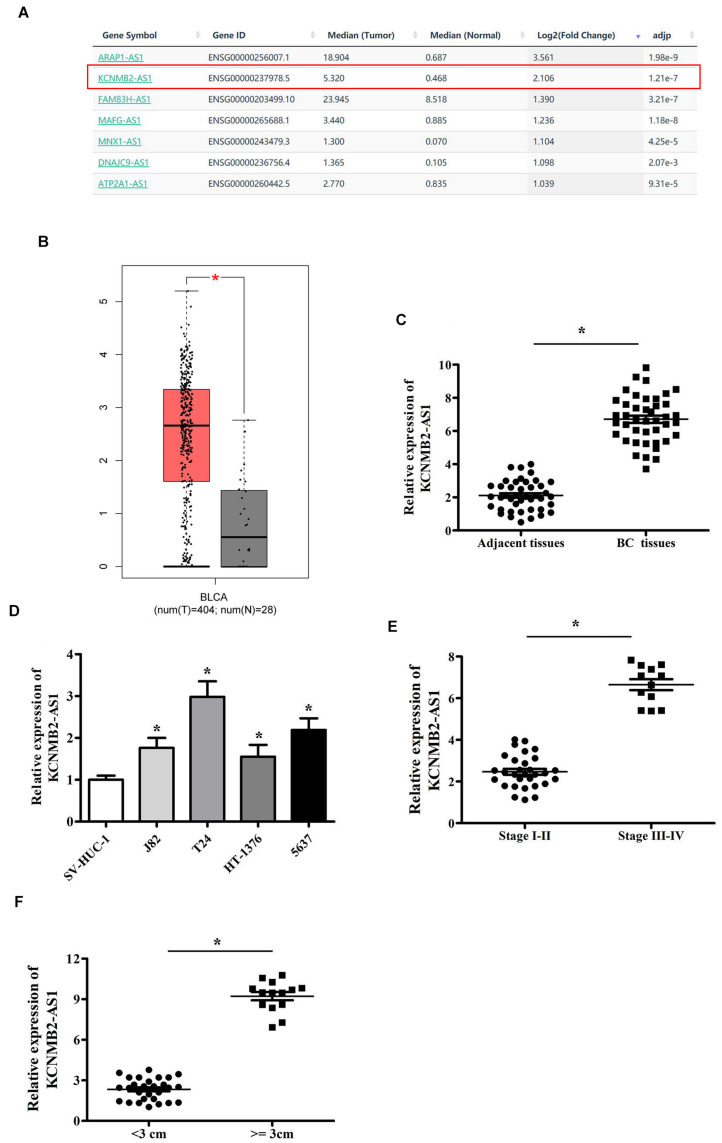
KCNMB2-AS1 was overexpressed in BC. **(A,B)** KCNMB2-AS1 upregulated in the BC tissues was chosen from GEPIA database. **(C)** Increased expression of KCNMB2-AS1 in BC tissues compared with adjacent non-tumor tissues. **(D)** KCNMB2-AS1 was upregulated in BC cell lines. **(E)** Upregulated expression of KCNMB2-AS1 was associated with advanced stage. **(F)** KCNMB2-AS1 expression significantly increased in the group with larger tumor size. Data were showed as mean ± standard deviation. *P < 0.05.

GEPIA database was utilized to select the upregulated lncRNA in BC, higher expression of KCNMB2-AS1 was observed in BC tissues than that in adjacent non-tumor tissues (Figures 1A,B). We found that KCNMB2-AS1 expression was significantly higher in BC tissues than in the adjacent tissues (Figure 1C). KCNMB2-AS1 expression in BC cell lines was notably higher than that in human normal urothelial cell line SV-HUC-1 (Figure 1D). In addition, we found that high levels of KCNMB2-AS1 were positively correlated with tumor size and TNM stage (Figures 1E,F).

### KCNMB2-AS1 Promoted BC Cell Proliferation, Migration and Invasion

**FIGURE 2:**
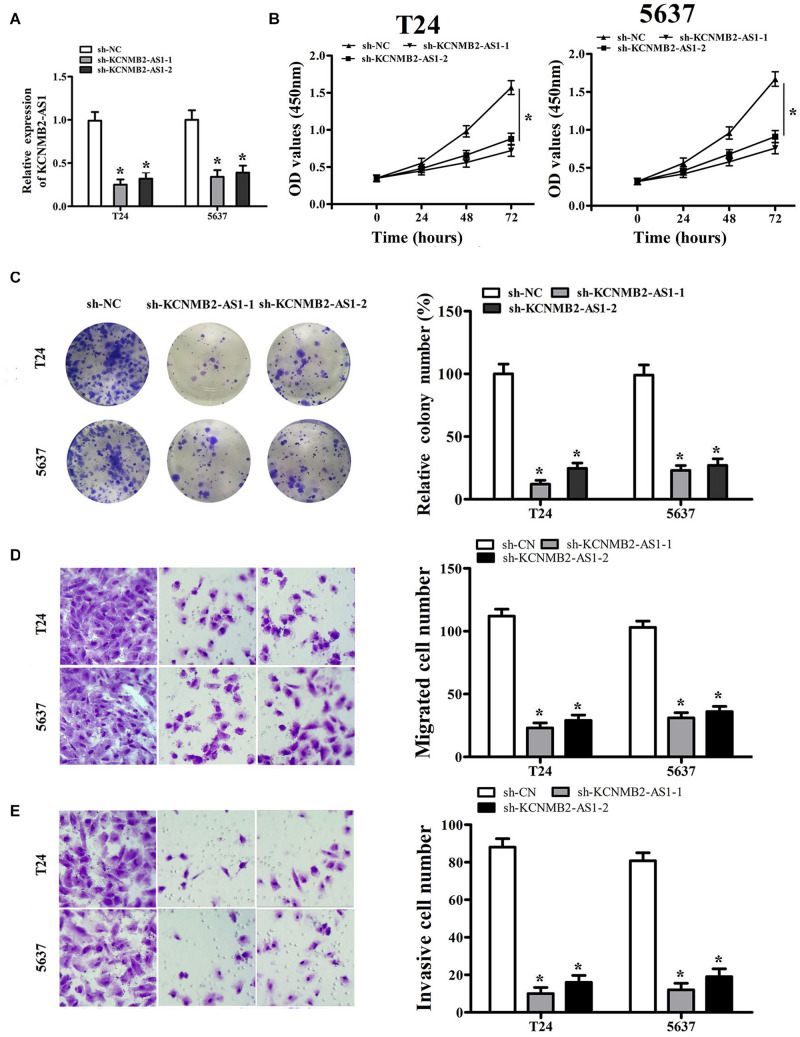
KCNMB2-AS1 promoted BC cell proliferation, migration, and invasion. **(A)** The efficiency of KCNMB2-AS1 knockdown was detected by qRT-PCR. **(B,C)** Cell proliferation was assessed in T24 and 5,637 cells by CCK8 assay and colony formation assay. **(D,E)** Transwell migration and invasion assays were performed after KCNMB2-AS1 knockdown. Data were shown as mean ± standard deviation. The experiment was repeated three times. *P < 0.05.

To investigate the biological role of KCNMB2-AS1 in BC cells. we silenced KCNMB2-AS1 expression in T24 and 5,637 cells using KCNMB2-AS1 shRNA (Figure 2A). CCK-8 assay and colony formation assay showed that the cell proliferation was remarkably declined after KCNMB2-AS1 knockdown (Figures 2B,C). Transwell assay demonstrated that sh-KCNMB2-AS1 inhibited T24 and 5,637 cell migration and invasion (Figures 2D,E).

### KCNMB2-AS1 Served as miR-374a-3p Sponge

**FIGURE 3:**
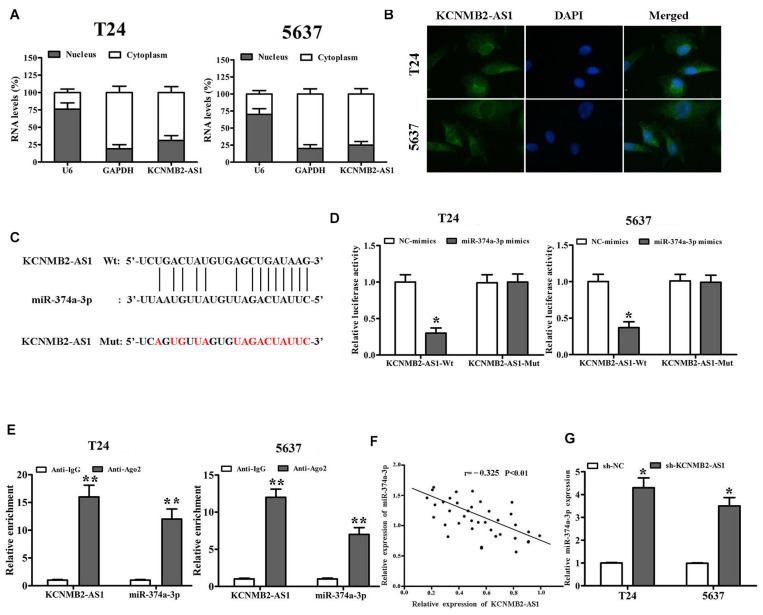
KCNMB2-AS1 was a sponge for miR-374a-3p in BC. **(A,B)** Subcellular fractionation and RNA FISH assays (×400) were used to confirm that KCNMB2-AS1 was located mainly in the cytoplasm. **(C)** Binding sites between KCNMB2-AS1 and miR-374a-3p were predicted by starBase. **(D,E)** Luciferase reporter assay and RIP assay indicated that KCNMB2-AS1 could bind with miR-374a-3p. **(F)** Pearson correlation was conducted to explore the correlation between miR-374a-3p and KCNMB2-AS1 in BC tissues. **(G)** miR-374a-3p expression was increased after KCNMB2-AS1 knockdown. Data were showed as mean ± standard deviation. The experiment was repeated 3 times. *P < 0.05. **P < 0.01.

We further investigated the regulatory mechanism of KCNMB2-AS1. Subcellular fractionation assay and RNA FISH showed that KCNMB2-AS1 was mainly localized in the cytoplasm of BC cells (Figures 3A,B). Given that LncRNAs regulate the expression of target genes by sponging miRNA. We found that miR-374a-3p had binding sites for KCNMB2-AS1 by using Starbase database^1^ (Figure 3C). Dual luciferase reports assay was performed to verify the potential combination between KCNMB2-AS1 and miR-374a-3p. MiR-374a-3p mimics remarkably attenuated the luciferase activity of KCNMB2-AS1-Wt compared with negative control groups (Figure 3D). RIP assay showed that KCNMB2-AS1 and miR-374a-3p were notably enriched in Ago2 groups compared to control IgG groups (Figure 3E). Moreover, we also observed that the expression of miR-374a-3p was negatively correlated with that of KCNMB2-AS1 from Pearson correlation analysis (Figure 3F). miR-374a-3p was significantly upregulated in cells transfected with sh-KCNMB2-AS1 (Figure 3G), indicating the negative regulation of KCNMB2-AS1 on miR-374a-3p.

### miR-374a-3p Targeted S100A10 in BC

**FIGURE 4:**
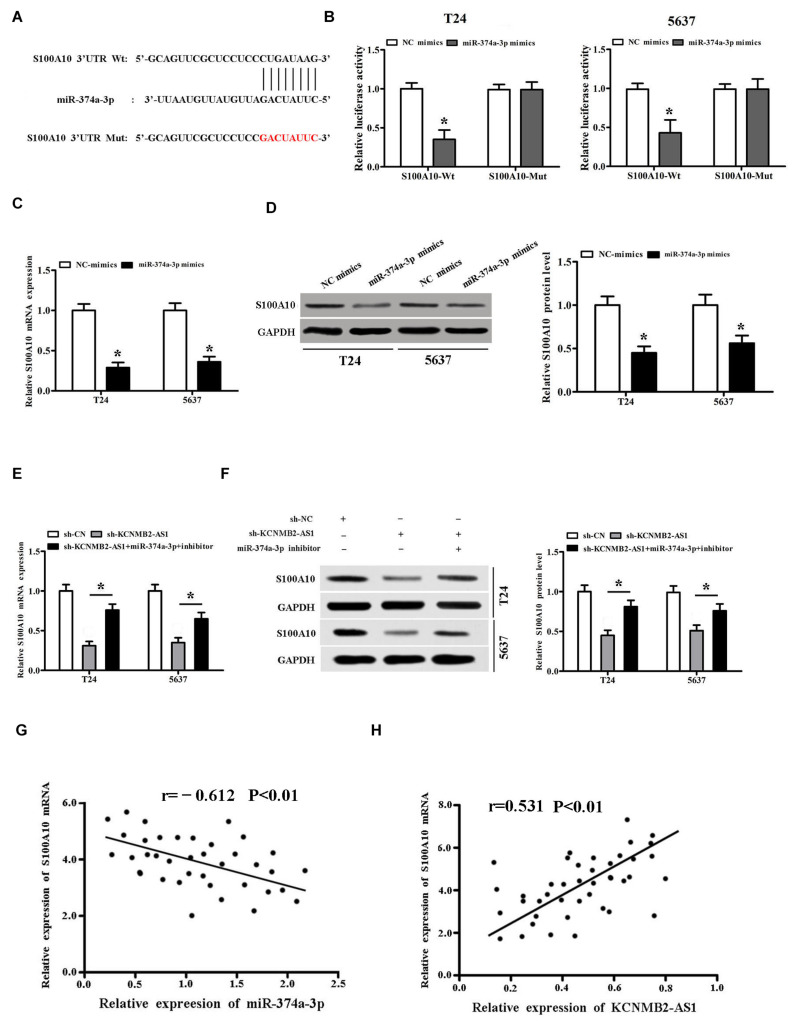
miR-374a-3p targeted S100A10 in BC. **(A)** TargetScan predicted that S100A10 had a binding site for miR-374a-3p. **(B)** Dual luciferase report assay validated the interactions among miR-374a-3p and S100A10 in BC cells. **(C,D)** S100A10 expression was inhibited by miR-374a-3p mimics. **(E,F)** miR-374a-3p inhibitor reversed the expression of S100A10 inhibited by sh-KCNMB2-AS1. **(G,H)** Pearson’s correlation analysis was conducted to evaluate the correlation of S100A10 and miR-374a-3p or KCNMB2-AS1 in BC tissues. Data were showed as mean ± standard deviation. The experiment was repeated 3 times. *P < 0.05.

According to TargetScan^2^ predictions, we found that miR-374a-3p could bind to the 3′-UTR region of S100A10 (Figure 4A). Luciferase activity assay showed that the luciferase activity of S100A10-Wt was inhibited by miR-374a-3p mimics, while S100A10-Mut not affected (Figure 4B). RT-PCR and Western blot results indicated that S100A10 expression was inhibited by miR-374a-3p mimics (Figures 4C,D). Moreover, we found that sh-KCNMB2-AS1 inhibited S100A10 expression, whereas miR-374a-3p inhibitor restored the expression of S100A10 inhibited by sh-KCNMB2-AS1 (Figures 4E,F). Pearson’s correlation analysis showed that there was an inverse correlation between miR-374a-3p and S100A10, and KCNMB2-AS1 was positively related to S100A10 in BC tissues (Figures 4G,H).

### KCNMB2-AS1 Promoted BC Progression Through Regulating miR-374a-3p/S100A10

**FIGURE 5:**
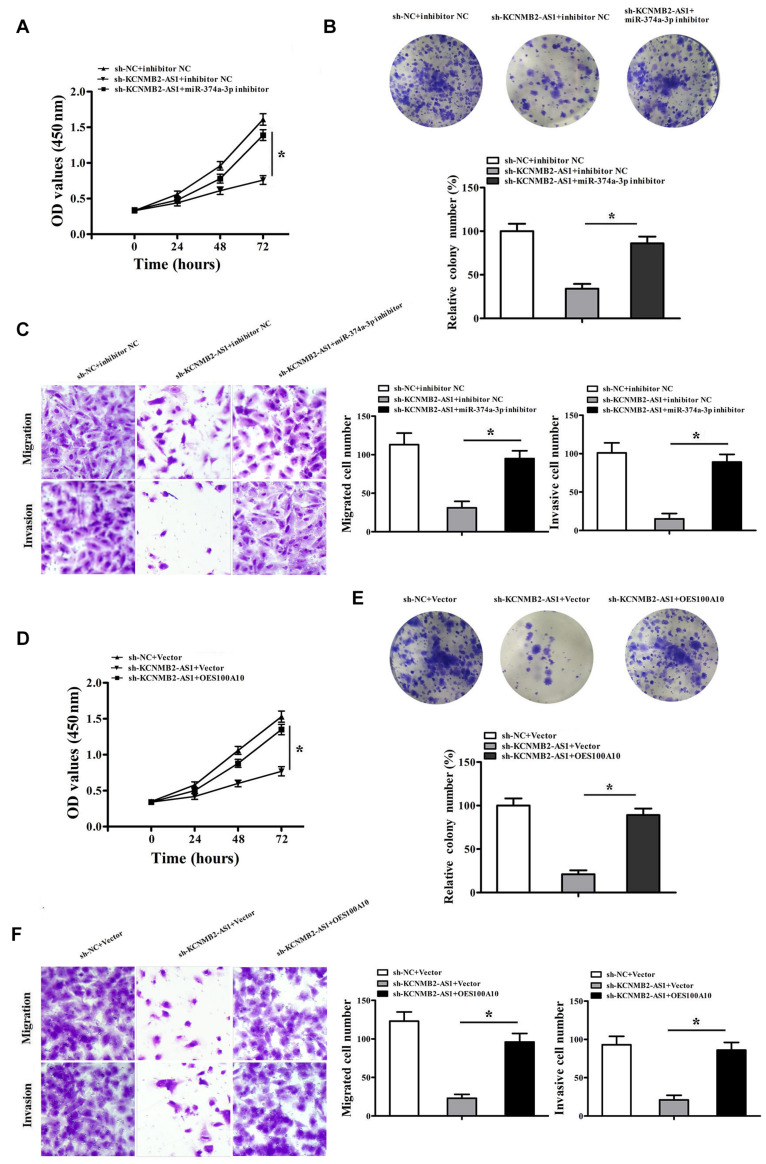
KCNMB2-AS1 promoted BC progression through miR-374a-3p/S100A10 axis. T24 cells were transfected with sh-NC + inhibitor NC, sh-KCNMB2-AS1 + inhibitor NC or miR-374a-3p inhibitor + sh-KCNMB2-AS1. **(A,B)** CCK8 and colony formation assay were performed. **(C)** Migration and invasion assay was performed. T24 cells were transfected with sh-NC + Vector, sh-KCNMB2-AS1 + Vector, sh-KCNMB2-AS1 + (overexpression, OE) OES100A10. **(D,E)** CCK8 and colony formation assay was performed. **(F)** Migration and invasion assay was performed. Data were shown as mean ± standard deviation. The experiment was repeated three times. *P < 0.05.

To investigate whether KCNMB2-AS1 promoted BC cell proliferation, migration and invasion through miR-374a-3p/S100A10 axis. Then CCK8 assay, colony formation assay, transwell assays and western blot were performed. We found that KCNMB2-AS1 knockdown inhibited proliferation, migration and invasion of T24 cells, whereas miR-374a-3p inhibitor reversed the effects of sh-KCNMB2-AS1 (Figures 5A–C). Furthermore, we restored the expression of S100A10. Results showed that S100A10 overexpression rescued the abilities of proliferation, migration and invasion in KCNMB2-AS1-depleted T24 cells. (Figures 5D–F). Taken together, these results demonstrated that KCNMB2-AS1 regulates BC progression through miR-374a-3p/S100A10.

### Knockdown of KCNMB2-AS1 Inhibited BC Progression *in vivo*

**FIGURE 6:**
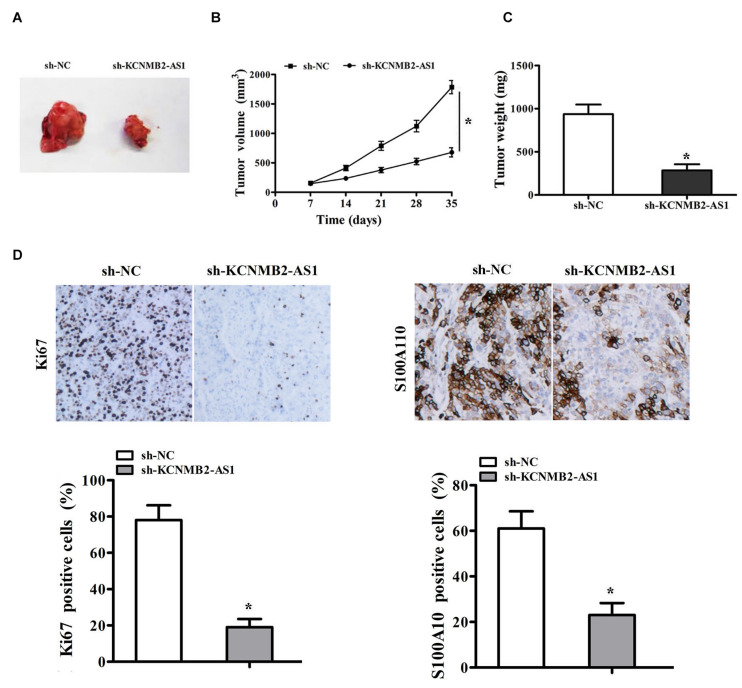
KCNMB2-AS1 knockdown inhibited BC cell growth *in vivo.* T24 cells transfected with sh-KCNMB2-AS1 or sh-NC were subcutaneously injected into nude mice to establish xenograft models. **(A)** Representative images of tumors. **(B,C)** The volume and weight of tumor xenografts. **(D)** Immunohistochemistry analysis of Ki-67 and S100A10 protein levels. Data were showed as mean ± standard deviation. Tumor volume at different time points was analyzed by repeated measures ANOVA, *n* = 4 for mice upon each treatment. *P < 0.05.

To confirm the role of lncRNA KCNMB2-AS1 in bladder cancer progression *in vivo*, we injected T24 cells transfected stably with sh- KCNMB2-AS1 or control sh-RNA into mice. Tumor in the sh-KCNMB2-AS1 group was smaller than in sh-NC group (Figures 6A,B). The tumor weight was also found lighter in the sh-KCNMB2-AS1 group than the sh-NC group (Figure 6C). Immunohistochemistry (IHC) assay showed that Ki67 and S100A10 expression was significantly decreased in sh-KCNMB2-AS1 group (Figure 6D). These results suggested that KCNMB2-AS1 promotes BC cell growth *in vivo*.

## Discussion

More and more evidence revealed that lncRNAs play a pivotal role in the development and progression of various cancers (Bartonicek et al., 2016; Zhan et al., 2016; Qiao et al., 2019; Zhang W. et al., 2020). Up to now, more than 10 000 lncRNAs have been identified, only a small portion of lncRNAs has been functionally characterized. LncKCNMB2-AS1 has been identified a new oncogenic lncRNA and reported to promote cervical cancer tumorigenesis (Zhang Y. et al., 2020). However, the role of lncKCNMB2-AS1 in bladder cancer is still largely unknown. In the present study, we found a new BC related lncKCNMB2-AS1, which was found significantly overexpressed in BC tissues and cell lines. Moreover, the level of KCNMB2-AS1 was positive correlation with the tumor stage. We showed that KCNMB2-AS1 acts as oncogenic lncRNA and promotes the progression of BC. Our data demonstrated that KCNMB2-AS1 take part in the development and progression of BC and may be a potential therapeutic target.

It has been reported that lncRNAs exert biological functions in regulating genes that influence tumor cell proliferation, migration, invasion and apoptosis (Chen et al., 2017; Cao et al., 2020; Huang et al., 2020; Zeng et al., 2020). For instance, in bladder cancer, Liu et reported that lncRNA SPRY4-IT1 promote bladder cancer cells proliferation and metastasis (Liu et al., 2017). Lnc XIST promotes bladder cancer cell growth and metastasis by regulating miR-139-5p mediated Wnt/β-catenin signaling (Hu et al., 2017). In the present study, we found that the knockdown of KCNMB2-AS1 inhibited the proliferation, migration and invasion, migration of BC cells *in vitro*. *In vivo* experiments, we demonstrated that KCNMB2-AS1 knockdown suppressed bladder tumor growth. These findings confirmed the role of KCNMB2-AS1 in BC progression.

KCNMB2-AS1 has been showed to be predominantly localized in the cytoplasm (Zhang Y. et al., 2020). Our study found that KCNMB2-AS1 is a cytoplasmic lncRNA in bladder cancer cells. The competing endogenous RNA (ceRNA) network of lncRNA/miRNA/mRNA has been reported as an important mechanism (Lian et al., 2018; Yang et al., 2018). For example, lncRNA PEG10 promotes bladder cancer cell proliferation through regulating miR-134 (Jiang et al., 2019). Miao et reported that LINC00612 acts as ceRNA by sponging miR-590 to enhance the proliferation and invasion of bladder cancer cells (Miao et al., 2019). KCNMB2-AS1 has been proved to be served as a ceRNA by sponging miR-130b-5p and miR-4294 in cervical cancer (Zhang Y. et al., 2020). Yang et al. (2020) showed that KCNMB2-AS1/miR-374a-3p/ROCK1 axis promotes NSCLC progression. In the present study, we speculated that KCNMB2-AS1 might function as a ceRNA in BC. Using bioinformatics analysis, we found miR-374a-3p could be a promising target miRNA of KCNMB2-AS1. Moreover, we proved the direct binding of KCNMB2-AS1 and miR-374a-3p using duel luciferase report assay and RIP assay. The function experiments further validated that KCNMB2-AS1 regulate the BC cell proliferation and metastasis by sponging miR-374a-3p.

S100A10 has been shown to be an important cancer promoter (Saiki and Horii, 2019). showed that knockdown of S100A10 significantly reduced colorectal cancer cell proliferation, migration, and invasion (Shang et al., 2013). In lung squamous cell carcinoma, S100A10 upregulation is found to be associated with poor prognosis (Sato et al., 2018). In our study, biological information and dual luciferase reporter assay confirmed that miR-374a-3p directly targeted S100A10. We demonstrated that miR-374a-3p had a negative regulatory effect on S100A10 expression. Pearson’s correlation analysis showed that miR-374a-3p was negatively correlated with S100A10 mRNA expression and KCNMB2-AS1 was positively correlated with S100A10 mRNA expression. Rescue experiments showed that S100A10 overexpression significantly rescued the effects of KCNMB2-AS1 knockdown on proliferation, migration and invasion of BC cells. These results demonstrated that KCNMB2-AS1 promotes the progression of bladder cancer through miR-374a-3p/S100A10 axis.

In conclusion, Lnc KCNMB2-AS1 serves as a ceRNA of miR-374a-3p in upregulating the expression of S100A10, thus promoting bladder cancer progression. Our study suggests that lncKCNMB2-AS1 may be a potential prognostic biomarker and therapeutic target for BC.

## Data Availability Statement

The original contributions presented in the study are included in the article/supplementary material, further inquiries can be directed to the corresponding author/s.

## Ethics Statement

The studies involving human participants were reviewed and approved by all patients signed informed consents, this study was approved by the Ethics Committee of Wuhan No.1 Hospital. The patients/participants provided their written informed consent to participate in this study. The animal study was reviewed and approved by all experimental and animal care procedures were approved by the Animal Research Ethics Committee of Wuhan No.1 Hospital.

## Author Contributions

JZ conceived and designed the experiments. YH, YZ, RH, and CH performed the experiments. JZ wrote the manuscript. All authors contributed to data analysis and gave final approval of the version to be published.

## Conflict of Interest

The authors declare that the research was conducted in the absence of any commercial or financial relationships that could be construed as a potential conflict of interest.

## Footnotes

1 starbase.sysu.edu.cn/

2 www.targetscan.org
